# Generation of synthetic TSPO PET maps from structural MRI images

**DOI:** 10.3389/fninf.2025.1633273

**Published:** 2025-09-08

**Authors:** Matteo Ferrante, Marianna Inglese, Ludovica Brusaferri, Nicola Toschi, Marco L. Loggia

**Affiliations:** ^1^Department of Biomedicine and Prevention, University of Rome Tor Vergata, Rome, Italy; ^2^Department of Surgery and Cancer, Imperial College London, London, United Kingdom; ^3^Department of Computer Science and Informatics, School of Engineering, London South Bank University, London, United Kingdom; ^4^Department of Radiology, Athinoula A. Martinos Center for Biomedical Imaging, Boston, MA, United States; ^5^Department of Anesthesia, Critical Care and Pain Medicine, Massachusetts General Hospital and Harvard Medical School, Boston, MA, United States

**Keywords:** synthetic PET, U-Net, TSPO, neuroinflammation, separable convolutions

## Abstract

**Introduction:**

Neuroinflammation, a pathophysiological process involved in numerous disorders, is typically imaged using [^11^C]PBR28 (or TSPO) PET. However, this technique is limited by high costs and ionizing radiation, restricting its widespread clinical use. MRI, a more accessible alternative, is commonly used for structural or functional imaging, but when used using traditional approaches has limited sensitivity to specific molecular processes. This study aims to develop a deep learning model to generate TSPO PET images from structural MRI data collected in human subjects.

**Methods:**

A total of 204 scans, from participants with knee osteoarthritis (*n* = 15 scanned once, 15 scanned twice, 14 scanned three times), back pain (*n* = 40 scanned twice, 3 scanned three times), and healthy controls (*n* = 28, scanned once), underwent simultaneous 3 T MRI and [^11^C]PBR28 TSPO PET scans. A 3D U-Net model was trained on 80% of these PET-MRI pairs and validated using 5-fold cross-validation. The model’s accuracy in reconstructed PET from MRI only was assessed using various intensity and noise metrics.

**Results:**

The model achieved a low voxel-wise mean squared error (0.0033 ± 0.0010) across all folds and a median contrast-to-noise ratio of 0.0640 ± 0.2500 when comparing true to reconstructed PET images. The synthesized PET images accurately replicated the spatial patterns observed in the original PET data. Additionally, the reconstruction accuracy was maintained even after spatial normalization.

**Discussion:**

This study demonstrates that deep learning can accurately synthesize TSPO PET images from conventional, T1-weighted MRI. This approach could enable low-cost, noninvasive neuroinflammation imaging, expanding the clinical applicability of this imaging method.

## 1 Introduction

The translocator protein (TSPO) is an 18-kDa protein primarily expressed on the outer mitochondrial membrane of multiple cell types, and is implicated in multiple physiological and pathological processes (Nutma et al., 2021). It was initially identified as the “peripheral-type benzodiazepine receptor” (Braestrup and Squires, 1977). However, further studies have revealed that TSPO is extensively distributed throughout various organs in the body, including the brain. In the central nervous system, its expression levels are very low in healthy conditions but become dramatically upregulated primarily by microglia and/or astrocytes in the context of neuroinflammatory conditions. Because of these expression properties, as well of our ability to image this protein using molecular imaging techniques such as [^11^C]PBR28 positron emission tomography (PET) imaging, TSPO has been extensively investigated as an in-vivo biomarker for neuroinflammation in various neuropathologies, such as neurodegenerative, psychiatric, chronic pain and other conditions (Loggia et al., 2015; Alshelh et al., 2022; Guida et al., 2022; Albrecht et al., 2019). However, the clinical utility of TSPO PET is hampered by its high costs, radiation exposure, and infrastructure requirements. Magnetic Resonance Imaging (MRI), in contrast, offers a safer and more widely accessible alternative. Previous studies have shown that MRI can detect structural and metabolic alterations associated with neuroinflammation (Albrecht et al., 2016; Quarantelli, 2015).

**Figure 1:**
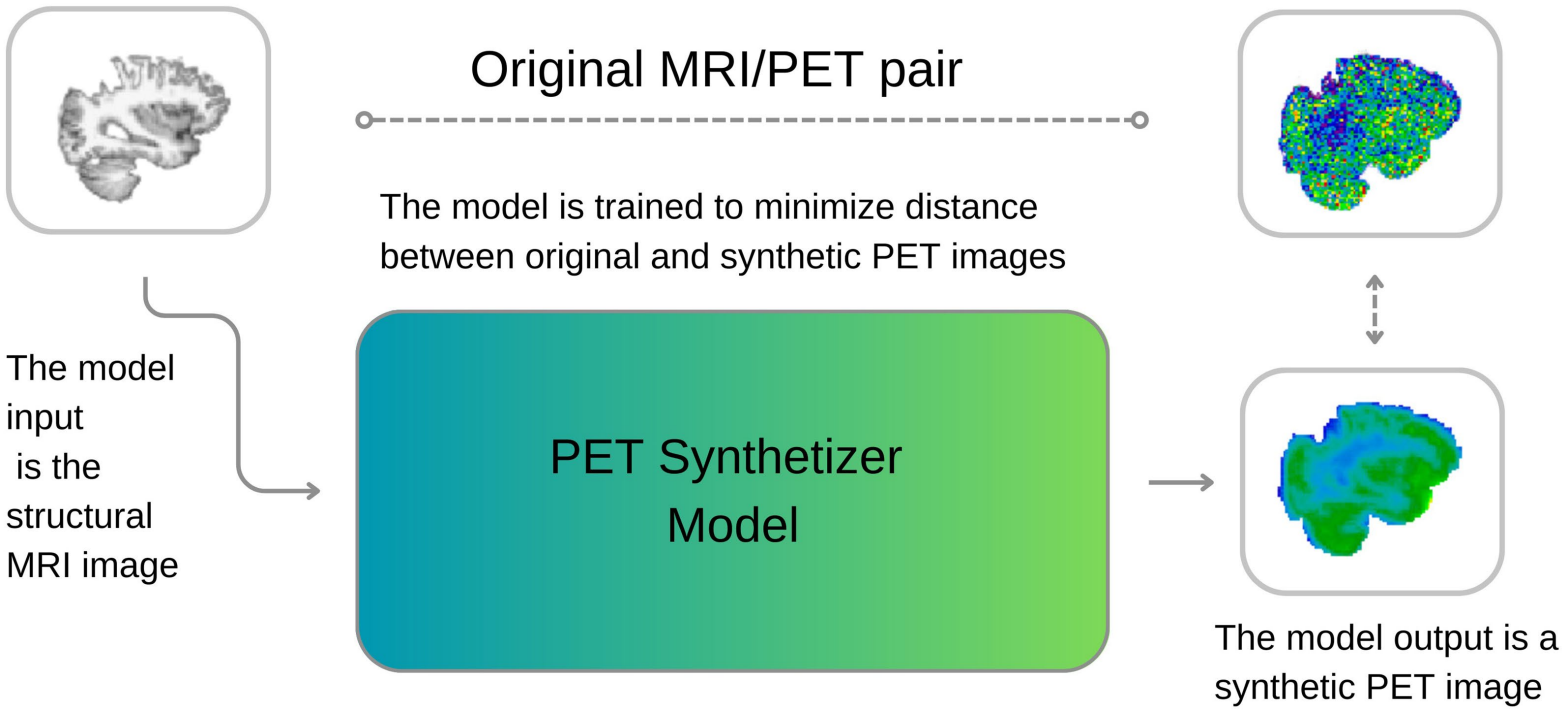
A visual description of our approach. During training, the model learns to synthesize PET images from MRI, minimizing the discrepancies between original and reconstructed images. During inference, the model can generate new synthetic PET images from structural T1w MRI images.

Leveraging this, multiple deep learning-based approaches have been developed to synthesize PET from MRI data to improve diagnostic performance, particularly in Alzheimer’s disease. Early approaches utilized 3D convolutional neural networks (CNNs) (Li et al., 2014) to learn nonlinear cross-modal mappings, outperforming conventional imputation techniques such as K-nearest neighbors. Models such as the 3D U-Net (Sikka et al., 2021) further improved spatial fidelity through skip connections and non-local feature aggregation. Generative Adversarial Networks (GANs) have since gained popularity for this task, with architectures like CycleGAN (Pan et al., 2021) employing cycle-consistency losses to learn bidirectional mappings, and pix2pix (Isola et al., 2017; Jung et al., 2018) using paired data with adversarial and L1 losses to generate sharper reconstructions. Additional innovations include normalization-aware adversarial U-Nets (Hu et al., 2019), which enhance learning through normalization strategies, and flow-based models such as Dual-Glow (Sun et al., 2019), offering efficient and invertible transformations. Recent models have introduced further refinements: BMGAN (Hu et al., 2020) incorporates bidirectional mappings and medical supervision; GANDALF (Shin et al., 2020) integrates classification feedback directly into the training process to improve diagnostic utility; and FREA-UNet (Emami et al., 2020) employs frequency-aware attention mechanisms to enhance PET realism and anatomical fidelity. Building on these advances, Theodorou et al. developed a 3D diffusion-based model (MRI2PET) that incorporates style transfer pre-training and a Laplacian pyramid loss to synthesize AV45-PET from T1-weighted MRI using unpaired data. Their approach boosted the AUROC for classification of Alzheimer’s disease, mild cognitive impairment, and cognitively normal individuals from 0.688 ± 0.014 to 0.780 ± 0.005 on the ADNI dataset (Theodorou et al., 2025). Similarly, Zhang et al. proposed BPGAN, a 3D GAN framework using a multi-convolution U-Net generator with gradient profile and structural similarity (SSIM) losses. Their model demonstrated improved PET synthesis quality and increased Alzheimer’s diagnostic accuracy by approximately 1% when synthetic PET was used in combination with MRI, compared to MRI alone (Zhang et al., 2022). Despite these advances, conventional MRI has low specificity for molecular-level processes. Inspired by the success of deep learning in medical image synthesis and translation, we here investigate the feasibility of generating TSPO PET images from T1-weighted MRI scans. Prior studies have shown the effectiveness of conditional GANs for generating CT from MRI (Wang et al., 2023), PET from MRI using E-GAN (Bazangani et al., 2022), and Florbetapir PET for Alzheimer’s diagnosis (Sikka et al., 2021). These methods have also enabled synthesis of high-dose PET from low-dose scans, reducing radiation exposure without compromising diagnostic quality (Sikka et al., 2021; Zhang et al., 2022; Sun et al., 2022). Our study aims to develop a deep learning model for PET image synthesis from T1-weighted MRI, using a 3D U-Net architecture (Figure 1) (Çiçek et al., 2016). We choose a 3D U-Net structure over, e.g., GANs or diffusion models due to its robust training behavior, ability to capture diverse data distributions, and suitability for limited training datasets. The model’s encoder-decoder structure facilitates the interpretation of imaging features and simplifies the synthesis process, making it a valid choice for PET synthesis from MRI. We therefore propose a tracer-specific modality conversion model based on U-Net, capable of generating synthetic brain PET images from T1-weighted MRI scans. This approach, if successful and validated, would offer a cost-effective, non-invasive method for imaging neuroinflammation, providing new insights into MRI features relevant to glial activation and related processes. Our work demonstrates the feasibility of synthesizing TSPO PET images from structural MRI images, hence significantly contributing to the non-invasive characterization of neuroinflammation.

## 2 Materials and methods

This section outlines our experimental setup. The code, developed in Python 3.9, leverages deep learning libraries such as Pytorch and Monai (Cardoso et al., 2022). All computations were conducted on a server with four NVIDIA A100 GPUs (80GB RAM each) and 2 TB of System RAM.

### 2.1 Dataset

A total of 204 scans, from participants with knee osteoarthritis (*n* = 15 scanned once, 15 scanned twice, 14 scanned three times), back pain (*n* = 40 scanned twice, 3 scanned three times), and healthy controls (*n* = 28, scanned once) were included for this study. They underwent simultaneous 3 T MRI and TSPO PET neuroimaging with [^11^C]PBR28. During cross validations (see below) when an individual had multiple scans, care was taken to segregate all scans from that individual either in the training or in the test split. Each participant received 9–15 mCi of [^11^C]PBR28 intravenously, and was scanned using a Siemens Biograph mMR for 90 min. From each scan, a Stanrdardised Uptake Value (SUV) map was reconstructed using 60–90 min post-injection data, which were attenuation-corrected using a T1-weighted structural data (multi-echo magnetization-prepared rapid acquisition with gradient echo (MPRAGE); TR/TE1/TE2/TE3/TE4 = 2530/1.64/3.5/5.36/7.22 ms, flip angle = 7°, voxel size = 1 mm isotropic) and the PseudoCT approach (Izquierdo-Garcia et al., 2014).

### 2.2 Pre- and post-processing

Our preprocessing pipeline, including alignment, skull stripping, and normalization, was performed using FSL (Jenkinson et al., 2012). Coregistration aligned both modalities to the same native space. Skull stripping, performed next, isolated the brain to eliminate non-brain signals. MRI volumes were normalized to a [0, 1] scale using robust scaling (1st and 99th percentiles) via MONAI, while PET volumes were normalized to SUV units based on individual minimum and maximum intensities. This approach harmonized intensities across subjects, thereby facilitating convergence in model training. Additionally, genotype data (high-affinity and mixed-affinity binder status), which affects [^11^C]PBR28 binding was used to adjust PET binding variability using FSL by regressing out the effect of genotype before further analysis (Owen et al., 2012). We implemented a 5-fold cross-validation strategy, dividing the 204 cases into five subsets. Each fold uses 80% of the data for training and 20% for testing, rotating across all subsets. To ensure data independence and avoid data leakage, we took special care when handling repeated acquisitions from the same subjects. Specifically, we ensured that all scans belonging to a given subject (i.e., repeated scans) were assigned to the same fold—either in the training, validation, or test set. Moreover, the distribution of different diagnostic groups was stratified across folds as evenly as possible to maintain balance during training and testing. The model’s objective (see “2.3 Model” section) was to synthesize TSPO SUV maps from structural MRI, while the reconstruction performance was evaluated against real PET images in the test set (see “2.4 Model Evaluation” section). This validation approach ensures robust assessment of the model’s capability to generalize from MRI to PET in our diverse patient cohort. To ensure a comprehensive and robust assessment of the synthesized PET image quality and the efficacy of our deep learning model (see below), a linear registration to MNI space and a nonlinear warping were applied to all T1 MRI images together with the true and synthesized PET images. This consistent registration enabled a thorough and standardized evaluation of the synthesized PET images against the true PET targets, using intensity, asymmetry, noise, and regional metrics as detailed below. Furthermore, under the hypothesis that the reconstruction process could filter out unstructured noise, we also compared reconstructed PET images to smoothed real PET images (4 mm full width half maximum (FWHM) kernel Gaussian filter).

### 2.3 Model

The 3D U-Net architecture was specifically adapted for the task of synthesizing PET images from MRI inputs. The core of the model’s design is a depthwise separable convolution-based encoder, which incorporates four downsampling layers. This approach minimizes the number of parameters in the model, thereby enhancing computational efficiency and facilitating the handling of three-dimensional data while reducing risk of overfitting. Each layer in the encoder and decoder pathways is comprised of 32 channels, optimizing the model’s capacity to capture a wide range of features in 3D medical images while conserving RAM usage.

Depthwise separable convolutions, a key component in our model, partition the standard convolution operation into two stages: depthwise convolutions and pointwise convolutions. This strategy, as discussed by Chollet (2017), drastically lowers the parameter count, thereby allowing for the inclusion of more channels in each layer without a proportional increase in computational demand. This technique is therefore instrumental in achieving high efficiency in feature extraction from volumetric data.

Moreover, the final layer of the encoder integrates a self-attention mechanism, thereby augmenting the model’s capability to understand global contextual relationships alongside local feature extraction. This enhancement is key for capturing long-range dependencies across the volumetric space of the input data and is hypothesized to aid in capturing underlying biological and anatomical structures.

On the decoder side, the architecture uses transpose convolutions for the purpose of upsampling, effectively restoring the spatial resolution that is reduced during downsapling. Skip connections, bridging corresponding layers in the encoder and decoder, are employed to reintroduce high-resolution details and features into the reconstructed images, ensuring that the latter can exploit the intricate details present in the original MRI scans. The training of the model was conducted over 50 epochs, using the Adam optimizer. The loss function we employed is a hybrid formulation that combines binary cross-entropy (BCE) and MSE, defined as follows in Equation 1:


(1) $$L=\alpha \cdot \mathrm{BCE}\;(y,\hat{y})+(1-\alpha )\cdot \mathrm{MSE}\;(y,\hat{y})$$

where *y* denotes the true PET images, $\hat{y}$ , represents the synthesized PET images, and *α* is a weighting factor that balances the contribution of each component to the total loss set as 0.5 in our experiments. This dual loss function is designed to ensure fidelity both in terms of the visual similarity and the voxel intensity distributions between the synthesized and true PET images, thus addressing both qualitative and quantitative aspects of the image synthesis task. In designing the 3D U-Net for PET image synthesis, several architectural choices were made to balance the expressiveness of the model and mitigate the risk of overfitting. First, the use of depthwise separable convolutions in both the encoding and decoding paths reduces the number of parameters by decoupling the spatial and channel-wise convolutions. This significantly lowers the model’s computational complexity while retaining the ability to extract meaningful spatial patterns, particularly important given the limited dataset. Additionally, group normalization was employed in all convolutional blocks, which is more stable for smaller batch sizes, helping to regularize training without the need for very large mini-batches. Moreover, multi-head self-attention layers were selectively added at higher resolutions to allow the model to capture long-range dependencies in the input data. These attention layers enhance the model’s ability to understand complex global relationships across volumetric inputs, which is critical for synthesizing detailed functional brain images. To further combat overfitting, skip connections are used between corresponding encoding and decoding layers, facilitating gradient flow and reintroducing high-resolution details into the output images. Finally, the model’s hybrid loss function (combining binary cross-entropy and mean squared error) is designed to encourage both structural and voxel-level fidelity in the synthesized image.

#### 2.3.1 Model architecture

The model is a 3D convolutional neural network based on a U-Net-like encoder-decoder structure, designed to process volumetric data of shape 96 × 96 × 96. The architecture incorporates residual learning, self-attention mechanisms, and optional depthwise separable convolutions to improve representational capacity while maintaining computational efficiency given the relatively limited training sample size. The overall structure consists of an initial projection layer, four levels of encoder and decoder blocks with skip connections, a central bottleneck, and a final reconstruction stage. Group normalization and Swish activation functions are used throughout the network to enhance training stability. The encoder comprises a series of DownBlock modules, each containing a residual block followed by an optional attention block. Between resolution levels, spatial downsampling is applied via strided 3D convolutions. The number of channels is progressively increased across resolution levels, following a multiplicative schedule. The bottleneck (MiddleBlock) processes the most compact feature representation through two residual blocks and an attention mechanism. The decoder mirrors the encoder in structure and employs UpBlock modules that concatenate the corresponding skip connections from the encoder. Upsampling is achieved via transposed 3D convolutions. The network concludes with a group normalization layer, a Swish activation, and a final 3D convolutional layer that projects the output back to a single channel. A Sigmoid activation constrains the final output to the [0, 1] range, suitable for applications such as segmentation or reconstruction. The complete architecture contains approximately 490,401 trainable parameters. Table 1 summarizes the major components of the model and their respective configurations.

**Table 1:** Summary of the 3D U-Net model architecture.

Module	Output shape	Layer type	Parameters
Input	−1, 1, 96, 96, 96	-	-
Image projection	−1, 32, 96, 96, 96	Conv3D	896
Encoder stage 1	−1, 32, 96, 96, 96	2 × Residual + Downsample	~4.2 K
Encoder stage 2	−1, 32, 48, 48, 48	2 × Residual + Downsample	~4.2 K
Encoder stage 3	−1, 32, 24, 24, 24	2 × Residual + Downsample	~4.2 K
Encoder stage 4	−1, 32, 12, 12, 12	2 × Residual + Attention	~4.2 K
Bottleneck	−1, 32, 12, 12, 12	Residual + Attention ×2	~8.5 K
Decoder stage 1	−1, 32, 12, 12, 12	Attention + Residual	~8.5 K
Decoder stage 2	−1, 32, 24, 24, 24	Upsample + Residual	~8.5 K
Decoder stage 3	−1, 32, 48, 48, 48	Upsample + Residual	~8.5 K
Decoder stage 4	−1, 32, 96, 96, 96	Upsample + Residual	~8.5 K
Final Conv	−1, 1, 96, 96, 96	Conv3D	1,089
Output	−1, 1, 96, 96, 96	Sigmoid	0
Total			490,401

### 2.4 Model evaluation

To rigorously assess our model’s performance in PET image synthesis, we employed a comprehensive framework comprised of various quantitative metrics, which are computed both at voxel level and at parcellation level. The latter, region-of-interest (ROI)-wise analysis was conducted using the cortical and subcortical Harvard-Oxford atlases (Desikan et al., 2006). Voxel-wise analysis incorporated the MSE to gage reconstruction accuracy, with percentage difference maps highlighting spatial error distribution. We also employed a metric called normalized difference (NormDiff) defined in Equation 2:


(2) NormDiff=(Reconstructed−True)(Reconstructed+True)

This metric evaluates relative errors, disregarding absolute intensity levels and has the advantage of being bounded between −1 and 1. Additionally, we included CNR was calculated due to its clinical relevance in diagnostic imaging. In this paper, the following definition of CNR was adopted as expressed in Equation 3:


(3) CNR=mean(ROI1)−mean(ROI2)std(ROI1+ROI2)

Since CNR varies locally, to produce a reliable global estimate for each image pair we proceeded as follows: we sampled ROI1 and ROI2 times (size: 4x4x3 voxels) at random 1,000 times in each PET image, computed CNR for each ROI, and compared the median CNR between the original and the reconstructed image.

### 2.5 Statistical analysis

To assess agreement between reconstructed and smoothed normalized SUV values, Bland–Altman analysis was performed, calculating mean differences and limits of agreement (±1.96 standard deviations). Pearson correlation coefficients with associated *p*-values were computed to evaluate linear relationships between reconstructed and smoothed SUVs region-wise. For group comparisons, subjects were divided into “Low SUV” and “High SUV” groups based on the median smoothed SUV value within each region. Differences in reconstructed SUV values between these groups were tested using the non-parametric Mann–Whitney U test. Statistical significance was considered at *p* < 0.05, and significant differences were annotated accordingly in the figures.

## 3 Results

**Figure 2:**
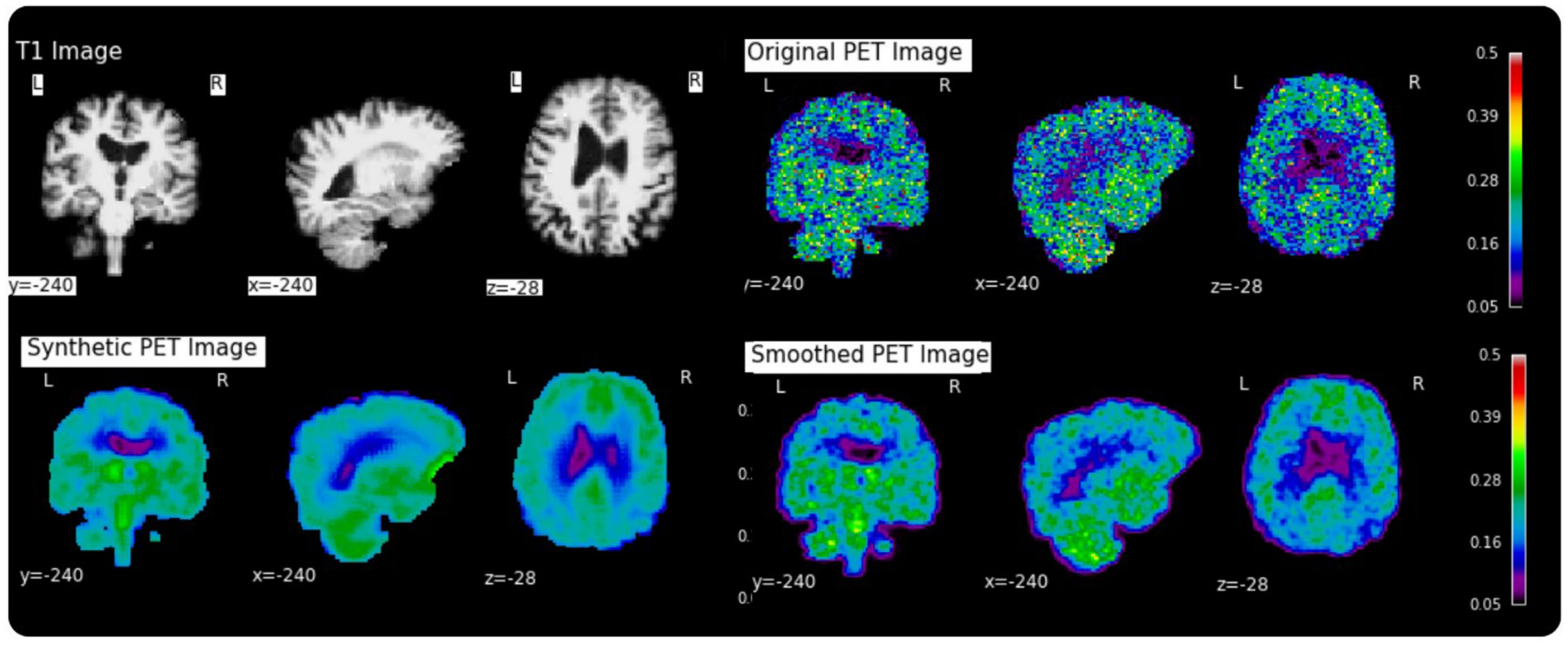
Examples of a random subject from the test set. The first row displays original intensity- normalized structural MRI and PET images, the second row presents our reconstructed images (left) and the original PET image post Gaussian smoothing (fwmh = 4 mm), (right).

**Figure 3:**
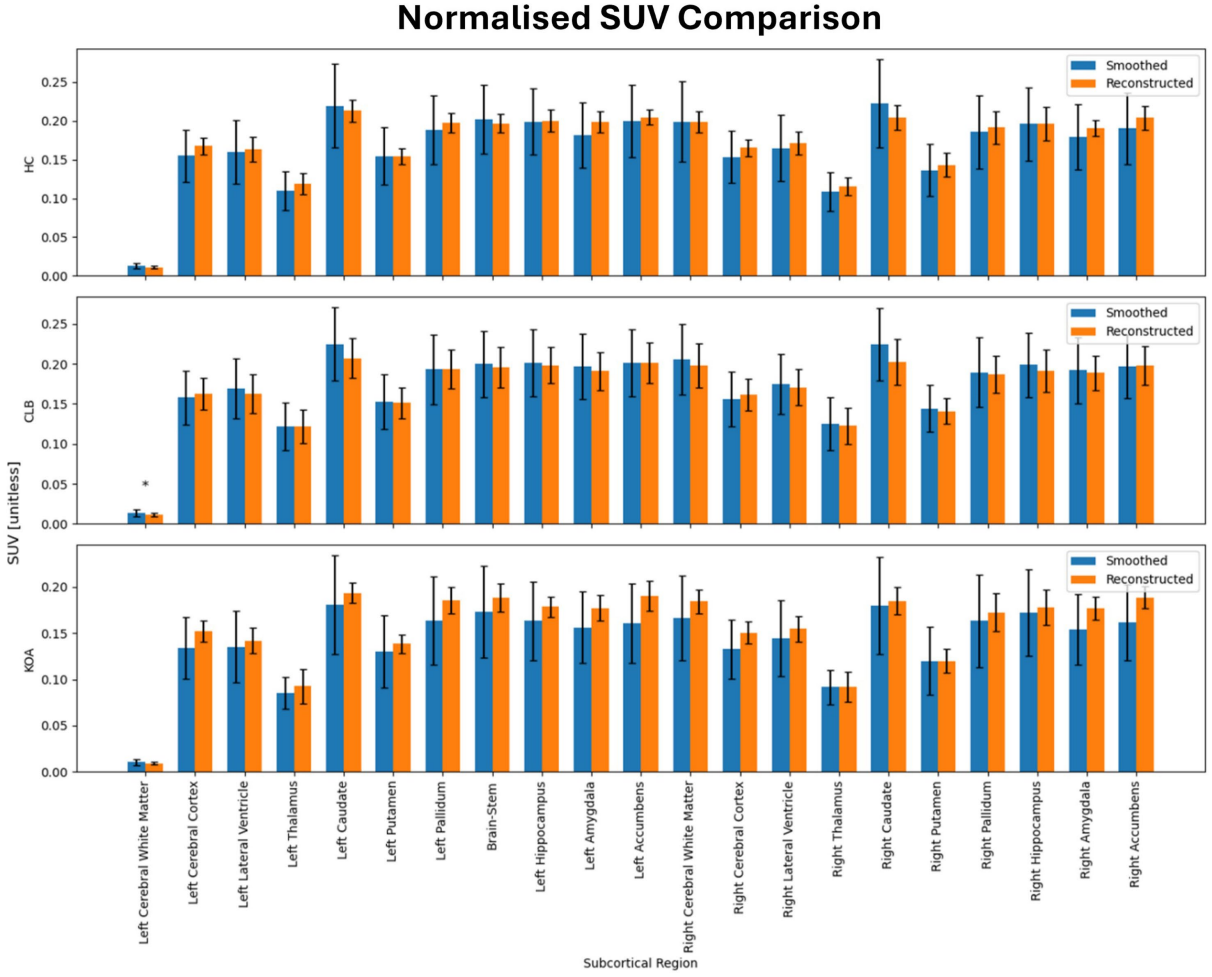
Results of ROI-wise analysis, which compared original to reconstructed PET images in terms of average normalized SUV in subcortical regions of healthy controls (HC) and subjects with back pain (CLB) and knee osteoarthritis (KOA). No statistically significant difference was found between original and reconstructed SUV in all the analyzed subcortical regions.

**Figure 4:**
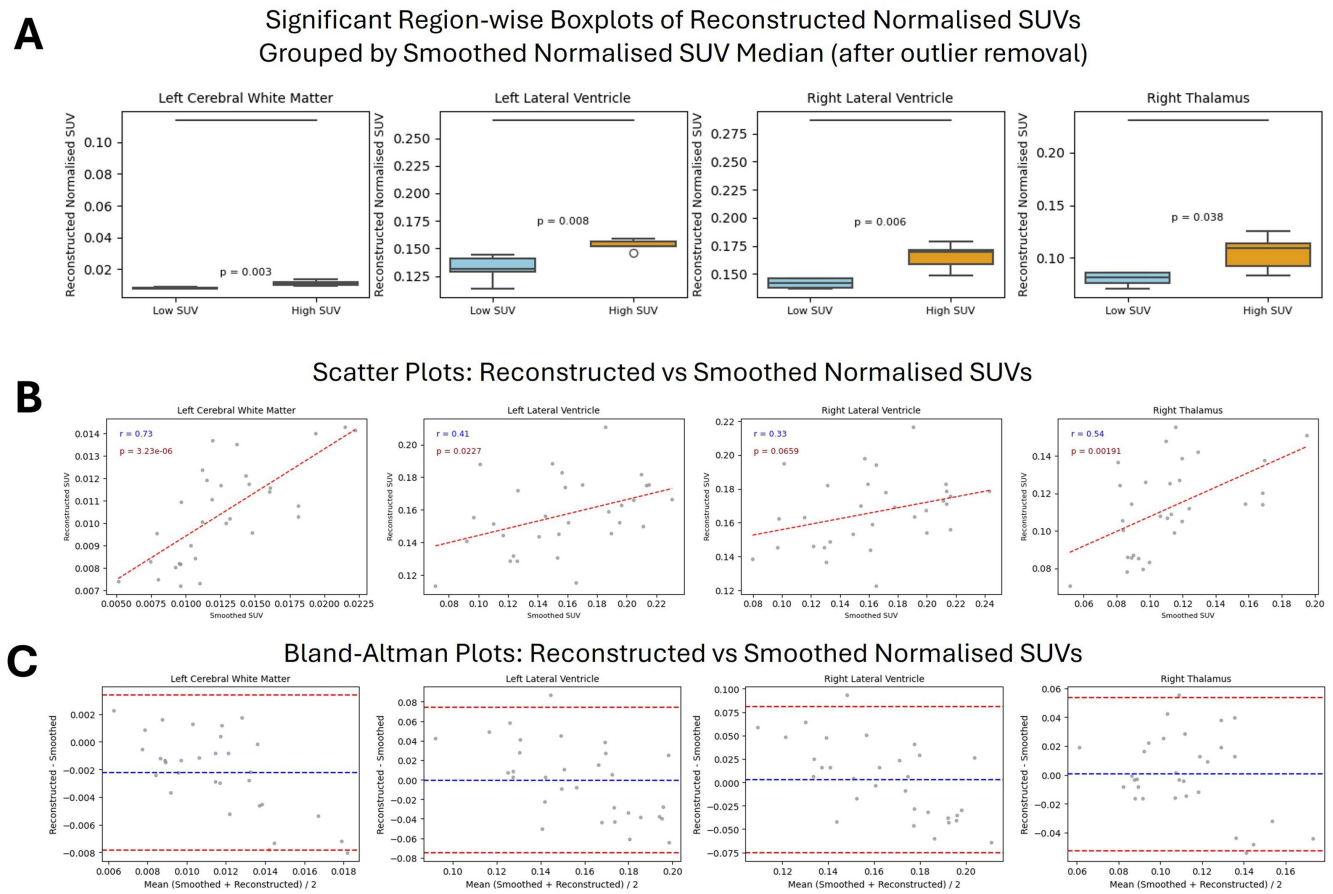
Region-wise comparison between Reconstructed and Smoothed (true) normalized SUV after outliers removal. **(A)** Region-wise boxplots of reconstructed normalized SUVs, grouped by whether corresponding smoothed SUV values are below (Low SUV Group) or above (High SUV Group) the regional median; **(B)** Scatter Plots of normalized reconstructed vs. smoothed SUV values. The Pearson correlation coefficient together with the associated *p*-value is shown on top of each plot. **(C)** Bland Altman Plots of normalized reconstructed vs. smoothed SUV values. The blue line indicates the mean difference between the two measurements, while the red lines represent the limits of agreement (±1.96 standard deviations from the mean difference).

**Figure 5:**
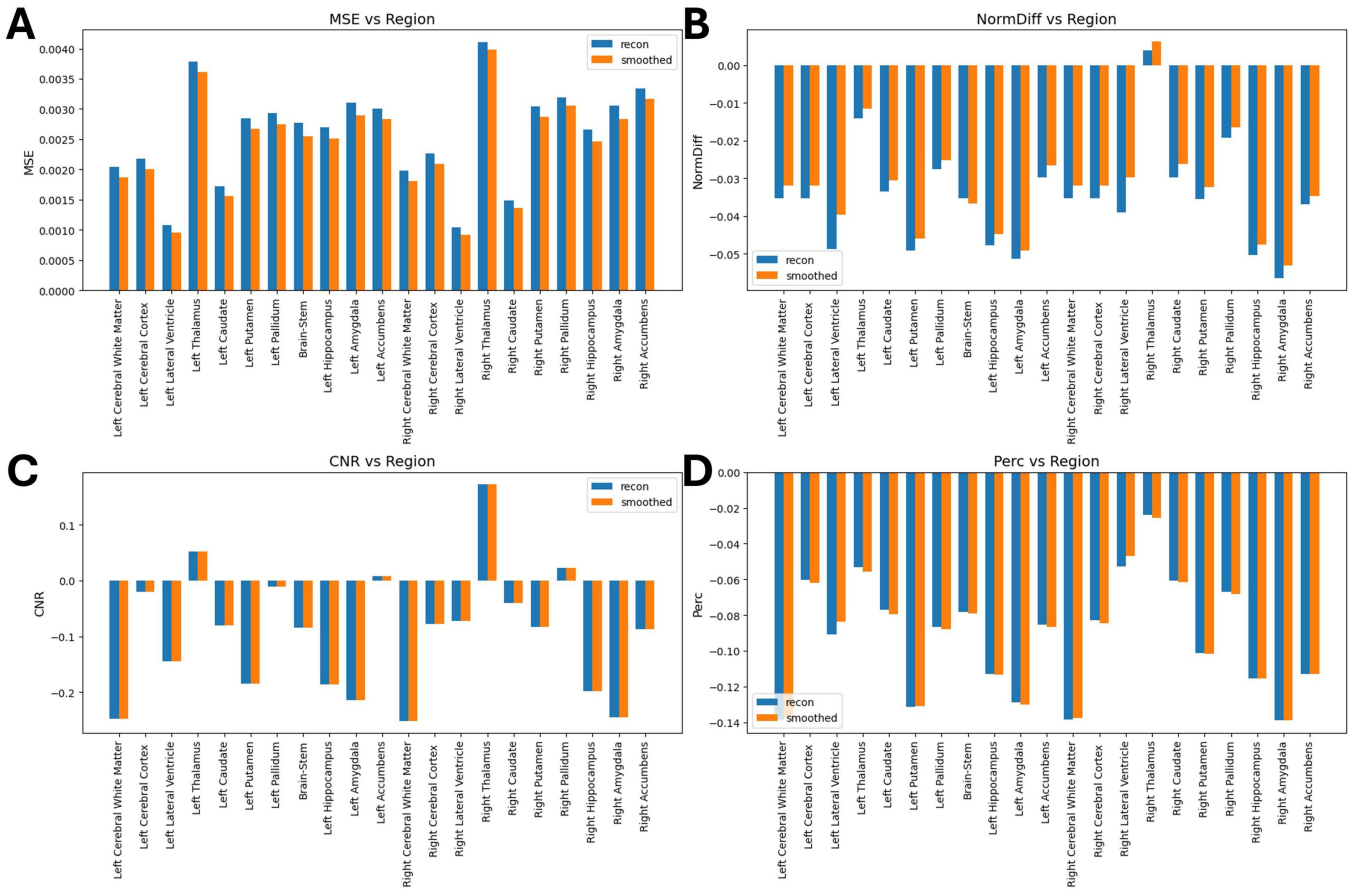
Results of ROI-wise analysis, which compared original to reconstructed PET images. **(A)** MSE comparison between synthetic images and original/smoothed. **(B)** Normalized Difference (NormDiff) between original and smoothed images. **(C)** Percentage difference analysis between original and smoothed images. **(D)** Median CNR comparison between original and smoothed images.

Table 2 shows the average normalized SUV evaluated in the original, smoothed original and reconstructed images and Figure 2 presents the result of the reconstruction for a representative subject from the test set. In particular, the first row displays original intensity- normalized structural MRI and PET images, while the second row presents our reconstructed images (left) and the original PET image post Gaussian smoothing. Figures 3, 4 show the results of the region of interest (ROI)-wise analysis of the average normalized SUV signal in the smoothed and reconstructed images. Except for a single statistically significant difference observed in the left cerebral white matter in the CLB group, no significant differences were found between the original (smoothed) and reconstructed PET data (Figure 3). Figure 4A shows boxplots of reconstructed SUV values stratified by median splits of the smoothed SUV within each region. After removing outliers, statistically significant differences between “Low SUV” and “High SUV” groups were observed only in a subset of regions: the left cerebral white matter, left thalamus, and the lateral ventricles. Among these, all showed *p*-values below 0.05 except for the right lateral ventricle, which demonstrated a trend-level difference. As shown in Table 3, several regions—including the left thalamus, caudate, pallidum, amygdala, and lateral ventricles—showed lower p-values when this same analysis was stratified by genotype (MAB or HAB) compared to the full cohort. This suggests that accounting for genotype reduces inter-subject heterogeneity and enhances the sensitivity to detect preserved SUV differences in the reconstructed images. Because the Ala147Thr polymorphism affects binding affinity (Owen et al., 2012), this observation further boosts the confidence that our method is genuinely sensitive to TSPO PET signal. None of the other regions exhibited statistically significant effects and are now shown. Figure 4B presents scatter plots of reconstructed versus smoothed SUV values for the same regions, highlighting positive associations and partial preservation of SUV magnitude information in the reconstructions. Figure 4C displays Bland–Altman plots for these regions, showing that most differences fall within ±1.96 standard deviations, indicating overall agreement. Notably, larger deviations were observed at the extremes of the SUV range, consistent with a regression-to-the-mean effect in the reconstruction. Together, these results suggest that while T1-based reconstruction using the applied 3D-UNet method may not fully capture all TSPO-binding related information, it retains some degree of normalized SUV magnitude information. Furthermore, the observed genotype-specific improvements in regional differentiation further support the relevance of stratified analyses in MRI-to-PET prediction frameworks. Figure 5 shows the normalized difference, mean squared error (MSE), percentage difference analysis and contrast-to-noise ratio metrics evaluated in the smoothed and reconstructed images. As also detailed in Table 4, we achieved a low MSE of 0.0034 ± 0.0010 (to be compared with the input and output values which span the interval [0, 1]) for raw synthesized PET, indicating minimal voxel-wise intensity error. Smoothing the synthesized PET images further reduces MSE. NormDiff, as defined in the 4.4 Section, showed a mean close to zero, confirming an unbiased reconstruction of PET SUV maps. Smoothing the synthesized images further lowered this measure, suggesting that our model could potentially also function as a signal enhancing smoothing filter. CNR, as defined in the 2.4 Section, calculations yielded similar average values across raw and smoothed synthesized images, demonstrating the preservation of spatial characteristics in synthesized PET.

**Table 2:** Normalized standardized uptake values (SUV) in the original, synthesized and smoothed PET images expressed as mean and standard deviation values.

Normalized SUV	Original image	Synthesized image	Smoothed image
Mean ± std	0.1419 ± 0.400	0.1548 ± 0.0452	0.1418 ± 0.0398

**Table 3:** Region-wise *p*-values for differences in reconstructed normalized SUV between low and high original smoothed and normalized SUV groups, computed across the entire cohort and stratified by genotype.

Region	ALL *p*-value	HAB *p*-value	MAB *p*-value
Left Cerebral White Matter	0.0047	0.0124	0.0159
Left Cerebral Cortex	0.6282	1	0.7302
Left Lateral Ventricle	0.0513	0.5493	0.4127
Left Thalamus	0.2949	0.1487	0.5556
Left Caudate	0.9452	0.0979	0.4127
Left Putamen	1	0.2451	0.4127
Left Pallidum	0.7308	0.8053	0.5556
Brain-Stem	0.9452	0.8053	0.4127
Left Hippocampus	0.6282	0.4595	1
Left Amygdala	0.8357	0.2451	0.7302
Left Accumbens	0.9452	0.9719	0.4127
Right Cerebral White Matter	0.6282	0.9719	0.1111
Right Cerebral Cortex	0.7308	0.9151	0.5556
Right Lateral Ventricle	0.366	0.647	0.5556
Right Thalamus	0.1375	0.0317	0.9048
Right Caudate	0.2343	0.3071	0.4127
Right Putamen	0.5338	0.3786	1
Right Pallidum	0.6282	0.7513	0.5556
Right Hippocampus	0.4452	0.7513	0.4127
Right Amygdala	1	1	0.4127
Right Accumbens	0.6282	0.8053	0.1905

For each subcortical region, we report the *p*-value from a Mann–Whitney U test comparing reconstructed SUV values between subjects with low versus high smoothed (true) SUV, as defined by a median split. *p*-values are shown for the entire dataset (ALL), and separately for subjects with MAB and HAB genotypes.

**Table 4:** Quantitative metrics comparing synthesized PET to raw and smoothed original PET images across the test set.

Evaluation metric	Mean ± Standard deviation
MSE	0.0034 ± 0.0010
MSE smoothed	0.0031 ± 0.0010
NormDiff	−0.0055 ± 0.0322
NormDiff smoothed	0.0010 ± 0.0311
CNR	0.0647 ± 0.2520
CNR smoothed	0.0647 ± 0.2521
PercDiff	−0.0219 ± 0.0658
PercDiff smoothed	−0.0226 ± 0.0645

MSE, Mean Squared Error; NormDiff, Normalized Difference; CNR, Contrast to Noise Ratio; PercDiff, Percentage Difference.

**Figure 6:**
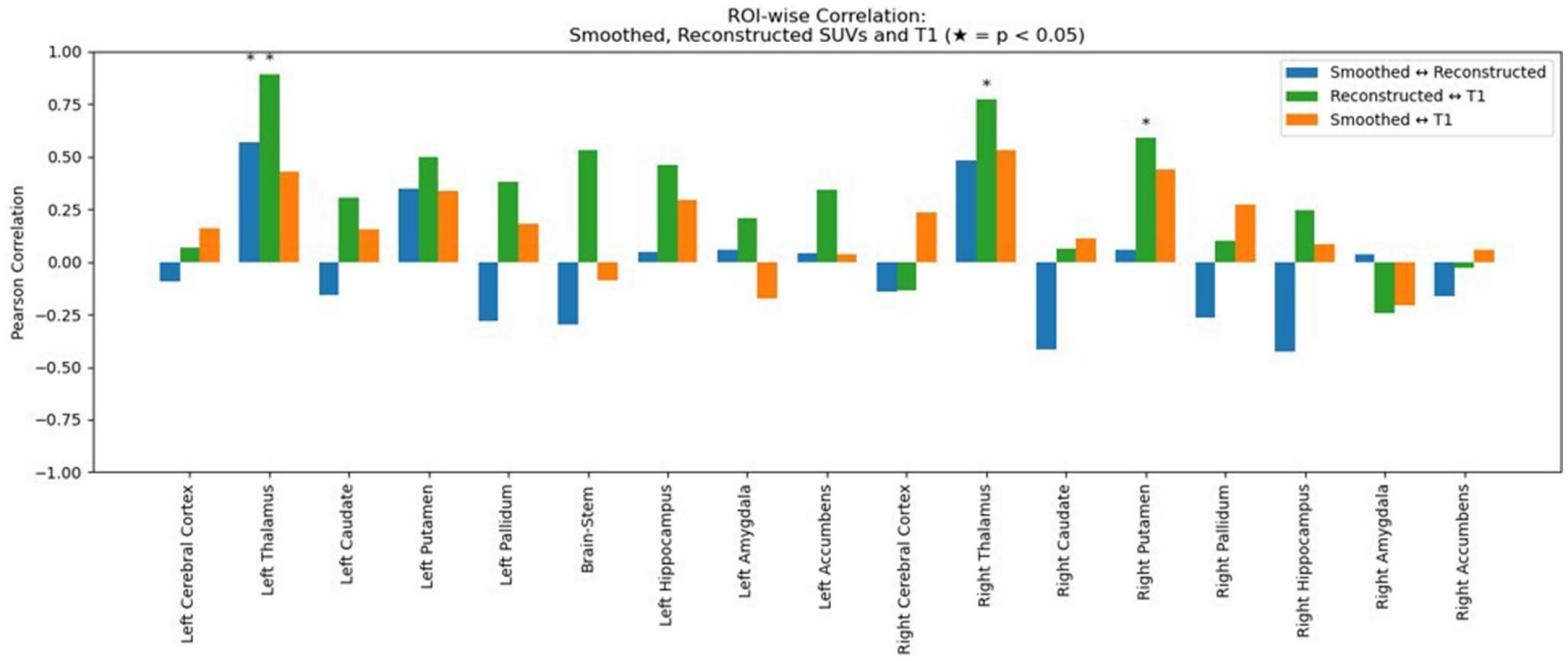
Region-wise Pearson correlation coefficients between image modalities. Blue bars show the correlation between smoothed (true) and reconstructed normalized SUV signal across subcortical regions. Green bars show the correlation between reconstructed PET and T1-weighted MRI signal and orange bars show the correlation between smoothed (true) normalized SUV and T1-MRI signal. Asterisks (*) indicate statistically significant correlations (*p* < 0.05).

**Figure 7:**
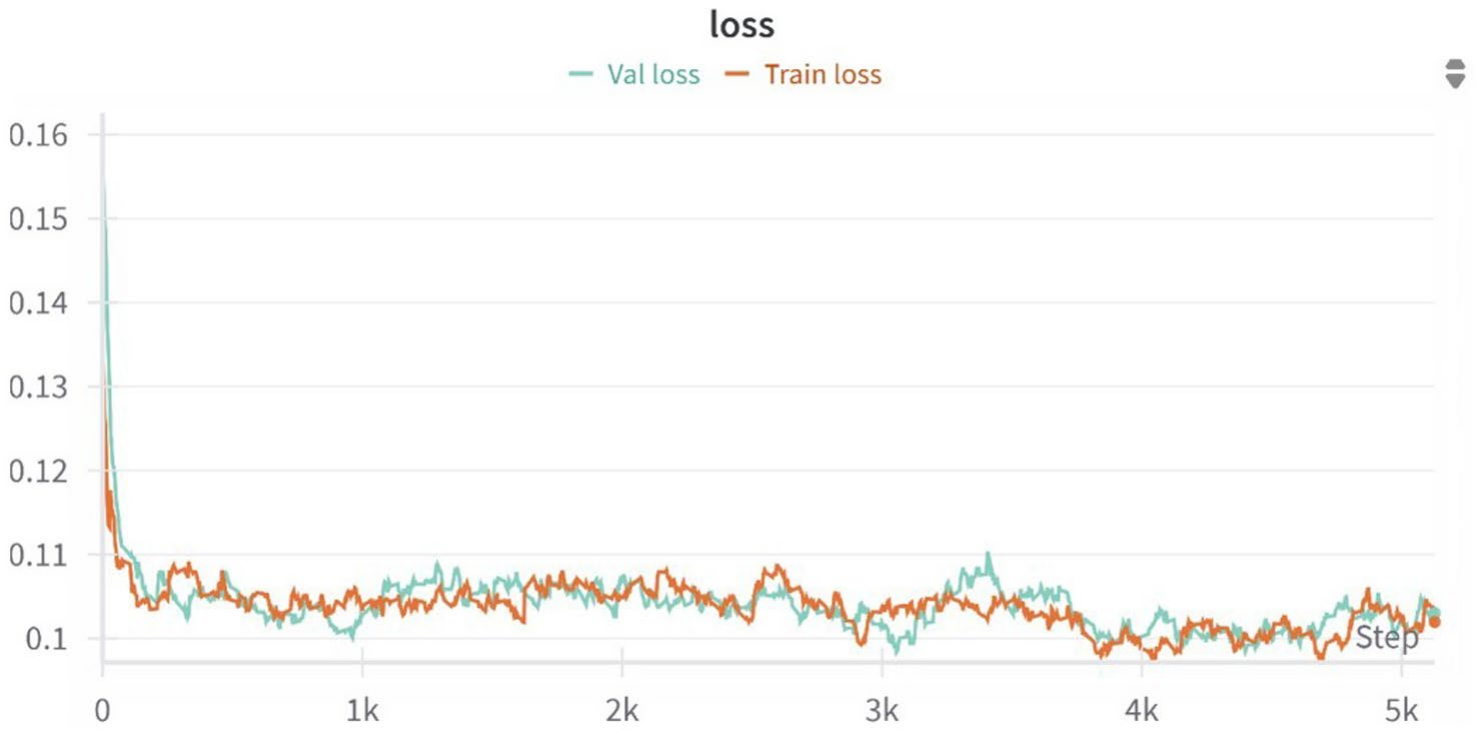
Training and validation loss curves over 5,000 optimization steps for the 3D U-Net model. The model rapidly converges within the first 500 steps and continues to improve gradually, with both training and validation losses stabilizing around 0.10, indicating no signs of overfitting and a good generalization performance across the dataset.

The mean percentage difference between synthesized and true PET was modest, with a slightly more negative bias observed after applying the smoothing filter. Additionally, we tested and compared the correlation (in terms of Pearson Coefficient) between reconstructed and smoothed SUV signal and reconstructed SUV and T1-MRI signal. As shown in Figure 6, our data show that the true TSPO PET signal exhibits generally low correlation with T1-weighted MRI values, suggesting it is not directly proportional to the anatomical signal. Together with that, the synthesized PET images also show relatively low correlations with T1, though slightly higher than the original PET, implying that the model does not simply replicate anatomical features but learns more complex patterns beyond direct structural mapping. Finally, the training and validation loss curves demonstrate a consistent decline and stabilization, indicating effective learning without evident overfitting (Figure 7). These results collectively underscore the model’s ability to synthesize PET images with high accuracy and minimal bias, reflected in low global error magnitudes and preserved intensity characteristics. The efficacy of the model is further evidenced by the consistent quality of PET synthesis across subsets, even after spatial normalization to MNI space, highlighting its robustness. Furthermore, we demonstrated that the model captures subject-specific TSPO binding patterns rather than general anatomical features.

## 4 Discussion

Neuroinflammation is being increasingly recognized for its role in a multitude of brain conditions; as such, monitoring this process may prove valuable in many clinical contexts. However, PET-based imaging of neuroinflammation -currently the best tool at our disposal- is hindered by high costs, and the need to expose patients to ionizing radiation. In contrast, MRI, which is safer and more widely available, could in principle offer an accessible alternative with its capability to detect neuroinflammation signal. We developed a deep learning model for PET image synthesis from T1-weighted MRI to transform widely available MRI scans into informative PET-like neuroinflammation patterns. While a direct comparison with previous PET synthesis studies is complicated by differences in target tracers, datasets, and evaluation strategies, we identified a relevant benchmark study by Pan et al. that reports percentage mean squared error (%MSE) values for several generative models trained on the ADNI dataset. Their architectures included GAN variants such as CycleGAN, VixGAN, L1GAN, and FGAN, with reported %MSEs ranging from 3.05% (standard GAN) to 1.80% (FGAN) (Pan et al., 2021). Our model achieved a %MSE of 3.4% for raw synthesized TSPO PET and 3.1% after smoothing. Although slightly higher, these values are in line with prior work, especially considering the different clinical targets (TSPO vs. amyloid or FDG), which likely exhibit greater inter-subject variability and lower anatomical congruence. Moreover, our use of a 3D U-Net offers a stable training performance, with its efficiency with small datasets and ability to capture diverse data distributions. In addition, the encoder-decoder design of the U-Net model allows an easy interpretation of the extracted imaging features simplifying the synthesis process, yet achieving comparable reconstruction accuracy. Our quantitative evaluation demonstrated high fidelity in PET reconstruction, with low voxel-wise error and minimal bias. We obtained a low voxel-wise mean squared error (0.0033 ± 0.0010) across all folds together with a median contrast-to-noise ratio of 0.0640 ± 0.2500. Region-wise analysis showed no significant differences between original and reconstructed PET, except in a single ROI, and the model preserved key intensity and contrast features. Importantly, reconstructed images were significantly more similar to the subject’s own original PET than to those of other individuals, indicating that the model captures subject-specific TSPO binding rather than generic anatomical patterns (Table 5). These findings support the model’s robustness and its potential for individualized PET synthesis from MRI. The results of this study have important implications for neuroinflammation imaging and chronic pain management. We introduced a novel and non-invasive method that could transform current diagnostic and therapeutic strategies for chronic pain conditions offering an opportunity to monitor neuroinflammation in chronic pain patients more frequently with a reduced cost. This could not only facilitate personalized treatment but also the acquisition of large-scale studies, potentially leading to a better understanding of the complex mechanisms of chronic pain and to the discovery of new therapeutic targets. Our model estimates spatial patterns of TSPO PET signal which align with existing research indicating variable which is thought to reflect glial density in different pain pathologies (Loggia et al., 2015), suggesting MRI’s promise in capturing such variations.

**Table 5:** Similarity metrics demonstrating subject-specific reconstruction of TSPO PET images.

Evaluation metric	Mean ± Standard deviation
MSE	0.0049 ± 0.0006
MSE smoothed	0.0044 ± 0.0006
NormDiff	−0.0441 ± 0.0576
NormDiff smoothed	−0.0440 ± 0.0582
PercDiff	−0.0423 ± 0.1250
PercDiff smoothed	−0.0401 ± 0.0852

MSE, Mean Squared Error; NormDiff, Normalized Difference; CNR, Contrast to Noise Ratio; PercDiff, Percentage Difference.

Average similarity metrics evaluated from the comparison between the reconstructed PET image of each subject and the original PET images of all other subjects in the dataset (excluding the subject’s own original PET used for reconstruction).

In this study, we used the SUV as the standardized measure of PET tracer uptake. However, the debate on the optimal PET quantification methods remains ongoing (Keyes, 1995) and different metrics are frequently employed across studies. For example, the SUV ratio (SUVr) could be explored in future applications to allow for a more precise analysis of tracer binding in the brain by comparing it with a reference region (Yoder et al., 2015). Additionally, moving beyond TSPO tracers to newer markers like P2X7, COX-2 could significantly enhance our understanding of microglial activation, paving the way for a multi-tracer PET synthesis approach (Yiangou et al., 2006) which would significantly enhance the availability of neuroinflammation assessment across centers. Although T1-weighted MRI does not directly capture molecular information such as TSPO binding, our model successfully reconstructs PET images with strong image-level similarity to the originals. This suggests that the model may be leveraging structural and volumetric features that are indirectly associated with neuroinflammatory processes, particularly in patient populations where such changes are more pronounced. TSPO expression reflects glial activation, which is not explicitly visible on conventional MRI; thus, the model’s performance likely relies on learning statistical correlations between anatomy and PET signal distributions. These findings highlight the need for caution in interpreting biological specificity. To improve physiological relevance, future research should explore the integration of additional MRI modalities—such as FLAIR, diffusion-weighted imaging, or perfusion MRI—which may better capture the underlying inflammatory processes, especially in regions like the cerebellum where discrepancies were observed.

Additionally, exploring the model’s applicability to other neuroinflammatory conditions beyond chronic pain could broaden its impact, making it a versatile tool in neurology and psychiatry.

### 4.1 Limitations and future directions

While our study demonstrates the feasibility of synthesizing TSPO PET images from structural MRI using a 3D U-Net, several limitations—both technical and clinical—must be acknowledged. First, the training dataset, though relatively large by simultaneous PET/MRI standards (n = 204), remains limited for deep learning applications and is derived from a single-center cohort. This may affect the model’s generalizability across scanners, imaging protocols, and patient populations. However, our deep model adopts modern U-Net components—such as residual blocks and attention layers—that are known to improve performance in volumetric tasks, while replacing standard convolutions with depthwise separable counterparts to reduce the total number of trainable parameters without sacrificing accuracy. This architectural design was guided by the need to balance expressive power with parameter efficiency, given that our training dataset is relatively limited in size. Furthermore, the dataset is not publicly available, restricting opportunities for external replication. However, it will be made available upon direct request to the senior authors and is expected to be publicly released in the near future. Together with that, future efforts will prioritize validation using multi-center, multi-vendor datasets and explore secure data-sharing approaches, such as anonymization or federated learning frameworks, to enhance reproducibility and robustness. Second, the model relies solely on T1-weighted MRI as input. While T1 captures structural anatomy, it lacks specificity for molecular or inflammatory markers like TSPO expression. The model likely learns statistical associations between anatomy and PET signal distributions rather than direct biophysical relationships. These associations could reflect subtle, non-obvious patterns or correlates of neuroinflammation—such as atrophy, regional vulnerability, or microstructural changes—that are not easily detectable by visual inspection but may still carry predictive value. We hypothesize that the model may detect true patterns of TSPO expression that are embedded in the MRI signal—though not directly visible to the human eye—by leveraging high-dimensional features or structural proxies. While this does not imply a direct mapping of physical signal between modalities, it suggests that information useful for predicting PET uptake might be inferable from structural context, especially when consistent patterns exist across subjects. To improve biological interpretability and reduce reliance on purely anatomical priors, future work should consider integrating additional MRI contrasts, such as FLAIR, diffusion-weighted imaging, or arterial spin labeling, which may offer greater sensitivity to inflammation, perfusion, or microstructural changes. Third, while the synthesized images show strong voxel-wise and regional similarity to ground-truth PET based on quantitative metrics, these measures do not fully capture clinical or biological interpretability, and whether they can meaningfully capture pathological processes remains to be evaluated. In fact, no structured reader studies or diagnostic decision-making tasks were performed. This reflects both the proof-of-concept nature of the study and current technical limitations. This limitation stems largely from the current signal normalization approach: both model input and output SUV images are scaled between 0 and 1 (crucial for improving model training and performance leading to faster convergence and better stability), which prevents direct quantitative comparisons between patients and controls. Addressing this issue in future work will be critical. This includes evaluating non-normalized outputs to assess whether group differences emerge in signal magnitude and/or spatial distribution. Additionally, incorporating expert radiologist assessments and task-specific performance benchmarks will be key to rigorously evaluating the clinical and diagnostic utility of the synthesized PET data. Finally, our use of standardized uptake value (SUV) as the reference target may not fully capture the complexity of TSPO PET quantification. Alternative measures such as SUV ratio (SUVr) or kinetic modeling may enhance physiological relevance in future studies. Together, these limitations point to key directions for further development: addressing the normalization step, expanding datasets, integrating multi-modal imaging inputs, improving model interpretability, and embedding clinical validation into the evaluation pipeline.

## 5 Conclusion

This study illustrates the successful synthesis of TSPO PET SUV images from structural MRI in chronic pain patients and healthy volunteers, marking a significant advance in non-invasive neuroimaging. The synthetic PET images generated by our model reproduce spatial signal distributions and related contrast properties which are extremely close to real PET scans.

In addition, synthesized PET volumes, while smoother than the original data, closely resembled Gaussian-smoothed PET scans. Given the conventional PET processing commonly includes smoothing, it is possible to hypothesize that our model inherently filters out unstructured noise, potentially enhancing signal-to-noise ratio and thereby the sensitivity for detecting subtle neuroinflammatory differences. In essence, our findings demonstrate that deep learning can effectively transform widely available MRI scans into informative PET-like neuroinflammation patterns. This approach promises to improve the noninvasive study of glial involvement in chronic pain and potentially other conditions, offering a novel perspective in medical imaging research.

## Data Availability

The data analyzed in this study is subject to the following licenses/restrictions: the datasets generated during and/or analyzed during the current study are available from the corresponding author on reasonable request. Requests to access these datasets should be directed to marco.loggia@mgh.harvard.edu.
